# A Review of *A Priori* Defined Oxidative Balance Scores Relative to Their Components and Impact on Health Outcomes

**DOI:** 10.3390/nu11040774

**Published:** 2019-04-03

**Authors:** Ángela Hernández-Ruiz, Belén García-Villanova, Eduardo Guerra-Hernández, Pilar Amiano, Miguel Ruiz-Canela, Esther Molina-Montes

**Affiliations:** 1 Department of Nutrition and Bromatology, Faculty of Pharmacy, University of Granada, 18071 Granada, Spain; angelahernruiz@correo.ugr.es (Á.H.-R.); ejguerra@ugr.es (E.G.-H.); 2 Nutrition and Food Science Doctorate Program (RD 99/2011), University of Granada, 18002 Granada, Spain; 3 Public Health Division of Gipuzkoa, Biodonostia Research Institute, Health Department, 20014 San Sebastian, Spain; epicss-san@euskadi.eus; 4 CIBER de Epidemiología y Salud Pública, CIBERESP, 28029 Madrid, Spain; 5 Department of Preventive Medicine and Public Health, University of Navarra, 31003 Pamplona, Spain; mcanela@unav.es; 6 Medicina Preventiva y Salud Pública, IdiSNA (Instituto de Investigación Sanitaria de Navarra), 31008 Pamplona, Spain; 7 CIBER Fisiopatología de la Obesidad y Nutrición (CIBEROBN), 28029 Madrid, Spain; 8 Genetic and Molecular Epidemiology Group, Spanish National Cancer Research Centre (CNIO), 28029 Madrid, Spain; memolina@cnio.es; 9 CIBER de Oncología, CIBERONC, 28029 Madrid, Spain

**Keywords:** review, oxidative stress, healthy diet, antioxidants, healthy lifestyle

## Abstract

Oxidative Balance Scores (OBSs) are tools that have emerged to evaluate the global balance of individuals’ oxidation—reduction status. The aim was to compare OBSs available in the literature regarding their characteristics and associations with chronic diseases in epidemiological studies. Studies that developed OBSs were searched in PubMed until August 2018. A total of 21 OBSs were identified. These OBSs presented different scoring schemes and different types of anti- and pro-oxidant components, including dietary factors (dietary intake and/or nutrient biomarkers), lifestyle factors, and medications. Most OBSs were based on over 10 components, and some included only dietary factors. Few considered weighted components in the score. Only three OBSs were validated as potential surrogates of oxidative balance through inflammation and OS-related biomarkers. Notably, all the OBSs were associated—to a varying degree—with a reduced risk of cardiovascular diseases, chronic kidney disease, colorectal adenomas, and different cancer types (colorectal and breast cancer), as well as with all-cause and cancer-related mortality. For other outcomes, e.g., prostate cancer, contradictory results were reported. In summary, there is a great heterogeneity in the definition of OBSs. Most studies are concordant in supporting that excessive OS reflected by a lower OBS has deleterious effects on health. Unified criteria for defining the proper OBSs, valuable to gauge OS-related aspects of the diet and lifestyle that may lead to adverse health outcomes, are needed.

## 1. Introduction

Oxidative stress (OS) is a multifactorial process caused by an imbalance between anti- and pro-oxidant components. Under normal physiologic conditions, human cells may restore the balance by upregulating antioxidant defense mechanisms [1]. In the event that these mechanisms are overwhelmed, un-neutralized free radicals prompt a series of reactions that can damage DNA, proteins, and lipids, leading to cell injury and death [2]. Various modifiable factors, such as diet, smoking, or medicines, are able to influence the OS process [3]. Thus, factors with antioxidant properties can halt the OS process or factors with prooxidant properties can boost the subsequent development of OS-related diseases such as cancer [4]. While a considerable body of evidence from basic science and animal studies support the role of OS as both an initiator and promoter of inflammation and disease occurrence [5], epidemiological studies have produced conflicting results regarding the impact that individual determinants of OS may have on health [6,7]. This lack of consistency could be attributable to the complex interplay between the numerous endogenous enzymatic mechanisms and exogenous modifiable factors involving multiple pro- and antioxidant factors by which OS may trigger the development of diseases. For example, among dietary factors, certain nutrients are presumed to function as antioxidants (e.g., carotenoids and tocopherols, vitamin C, flavonoids, PUFAs, and minerals: zinc, selenium, copper) or pro-oxidants (e.g., saturated fat, iron) [8], while smoking is a recognized pro-oxidant factor. Such interactions and correlations between these factors makes it difficult to ascertain the independent effects of oxidant or antioxidant factors on disease risk. 

A combined measure of multiple pro- and antioxidant exposures could be a more accurate indicator of the OS stress burden of an individual [9]. Oxidative balance scores (OBSs), account for dietary and lifestyle anti- and pro-oxidant factors. In these scores, antioxidant factors usually contribute positively whereas pro-oxidant factors contribute negatively, and therefore a higher OBS reflects a predominance of antioxidant relative to pro-oxidant exposures. The first OBS was constructed as a combination of intake of two dietary antioxidants (vitamin C and ß-carotene) and a single dietary pro-oxidant (iron) [9]. Although this OBS was inversely associated with a lower mortality risk in smokers, it was not considered to adequately represent the overall exposure to anti- and pro-oxidants. In this context, several OBSs have been created using multiple and different approaches, comprising not only dietary and lifestyle factors and medication but also biomarker components and genetic variants [10]. In addition, there are differences in the construction of these OBSs, regarding how the individual components are modeled and defined in the score, among others. 

A number of studies have also reported the association between the OBS and components thereof with different diseases, such as cancer [11,12], and also mortality [9]. However, while some OBSs have been associated with a lower risk of developing colorectal adenomas or lung cancer [11,13,14,15], others have failed to show such a risk-lowering effect for prostate cancer [12,16,17]. A critical evaluation of the definition and construction process of OBSs will shed some light on the capacity and utility of these scores to show how different factors modulate the occurrence of OS-related diseases. 

Our aim was to analyze and compare the characteristics of OBS published in the literature and to review the association of these OBSs with different health outcomes, especially chronic disease related with OS. Particular attention was paid to the review of OBS components and their capacity to modulate the balance between a pro- and antioxidant status in the body.

## 2. Materials and Methods 

### 2.1. Data Sources and Search Strategy

A comprehensive review was conducted by systematically searching in PubMed for all studies that developed an *a priori* OBS until 15 August 2018. Medical subject headings related to OBSs (Antioxidant, Oxidants, and Oxidative Stress) and other key terms (Antioxidant score, Prooxidant score, *a priori* oxidative balance score, and *a priori* oxidative balance) were considered. The search strategy was defined as: (Antioxidant (MeSH Terms) OR oxidants (MeSH Terms) OR oxidative stress (MeSH Terms) AND antioxidant score (All fields)) OR prooxidant score OR a priori oxidative balance score OR a priori oxidative balance. The search was limited to English and human studies. In addition, all references included in the selected articles were reviewed to retrieve studies on OBSs not found by the initial search.

### 2.2. Inclusion and Exclusion Criteria

Studies were eligible if they described the use of an *a priori* OBS defined by antioxidant and pro-oxidant components including dietary and lifestyle factors, biomarkers, and/or medication. Studies were first screened by title, then by abstract, and finally by reading the study in full. Two researchers (AHR and BGV) identified the studies and carried out the data abstraction. Disagreements were resolved by discussion with a third researcher (EMM). 

In a first phase, all studies (case-control or cohort studies) describing a certain OBS were included for the comparison of the characteristics of the OBSs. In a second phase, studies assessing the association between OBS and disease risk were considered. Outcomes of interests were OS-related determinants (e.g., obesity and inflammation), overall mortality, cause-specific mortality, and/or risk of cardio- and cerebrovascular diseases, cancer, or neurodegenerative diseases.

### 2.3. Data Extraction

Data extracted included first author, year of publication, study design, country, study population, duration of follow-up, main outcomes, covariates included for adjustment, main results and reported risk estimates, and the main characteristics and methodological features of each included OBS. 

## 3. Results

The search strategy retrieved 965 records. Twenty-one original articles on OBS were selected [9,10,11,12,13,15,16,17,18,19,20,21,22,23,24,25,26,27,28,29,30]. These OBSs presented differences regarding their composition and association with health outcomes.

### 3.1. Components of the OBSs

A detailed description of the OBSs focusing on their components and scoring systems is shown in Table 1. The number of components of the OBSs varied considerably among the scores, ranging between three and 20 components. There were five OBSs containing 13 components [16,19,22,25,27], four OBSs with 14 components [11,18,24,26], two OBSs with 12 components [10,15], two OBSs with 15 components [13,28], and two OBSs with eight components [17,29]. The remaining OBSs presented either 20 components [12], 16 components [23], 11 components [20], seven components [30], six components [21], or three components [9].

Since OBSs feature both antioxidant and pro-oxidant components, all the identified OBSs included a combination of these components, though with considerable variations among the OBSs. Interestingly, all OBSs included a higher number of antioxidant components than pro-oxidant components. Only five OBSs presented dietary biomarkers as surrogates of antioxidant or pro-oxidant nutrients, and also included lifestyle and medication components [10,11,18,22,25]. All other OBSs included dietary components with lifestyle components [13,16,17,23,28,29,30] or medication [27], or both lifestyle and medication components [10,11,12,15,18,19,20,22,24,25,26]. Only two OBSs were based on dietary components alone [9,21].

Table 2, Table 3 and Table 4 give details of the specific lifestyle factors, medication, dietary, biomarkers, and food components included in each OBS. Among dietary antioxidants, almost all OBSs included Vitamin C, β-carotene, and vitamin E; a significant proportion of OBSs included lycopene, lutein/zeaxanthin, and selenium, while fewer OBSs considered Vitamin B_9_ [19,21], retinol [30], vitamin D [19], zinc [12], calcium [19], and total catechin [17]. Concerning dietary pro-oxidants, alcohol intake and iron—only one OBS considered heme iron [17]—were among the components included most often, followed by PUFAs and SFAs, whereas fats in general were considered by one OBS [29]. There were four OBSs in total that assessed antioxidant and pro-oxidant status by biomarker components [11,18,22,25], and one that only accounted for antioxidant biomarkers [10]. The OBSs developed by Lakkur et al., 2014 [22] and Goodman et al., 2010 [18] considered the largest number of biomarker components. Only one of the OBSs took into account antioxidant (crucifers) and pro-oxidant (red meat) food components [16]. 

As regards lifestyle factor components, smoking was a key component for all but two OBS [21,27], and physical activity or obesity was deemed necessary in seven OBSs [13,22,23,25,28,29,30]. Both aspirin and NSAID representing medication components were considered in 11 OBSs [10,11,12,15,18,20,22,25,26,27]. Moreover, two OBS considered genetic variants of genes involved in the body’s antioxidant network [19,21].

**Figure 1:**
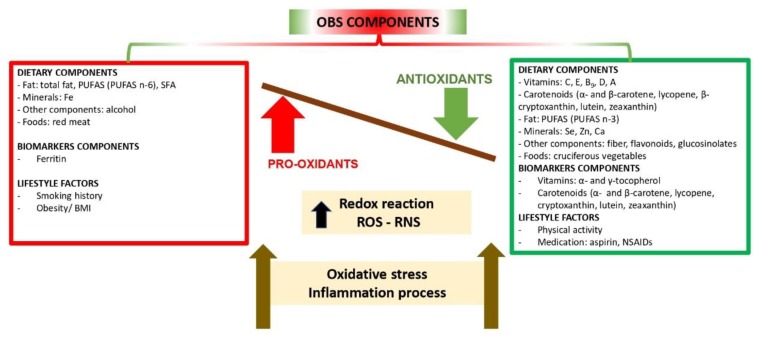
Components of the Oxidative Balance Scores (OBSs). Imbalance between the components in favor of a pro-oxidant state leads to OS and inflammation unless neutralized. ROS: Reactive Oxygen Species; RNS: Reactive Nitrogen Species; PUFAS: polyunsaturated fatty acids; NSAID: Non-Steroidal Anti-Inflammatory Drug; SFA: Saturated Fatty Acids.

The connecting relationships between all components considered in published OBSs are illustrated in Figure 1.

### 3.2. Scoring Systems

Table 5 describes the methodological criteria of each OBS. The defined ranges and the cutoff thresholds varied among the scores. There were either population-dependent cutoffs or predefined components for the majority of the categorical variable components. All but three OBSs [9,16,21] used both the population-dependent and predefined components. Regarding to the population-dependent components, they were divided into quantiles by medians [10,13,23] tertiles [9,11,15,18,19,20,22,24,25,26,27,30], quartiles [12,16,17,21,29] or even quintiles [16]. Certain components of some OBSs even applied sex-specific quantiles [15,20,24,27,29]. The predefined components in the categorical components, i.e., lifestyle factors and medication, were mostly divided into three pre-established groups, except in some OBSs that considered two [13,19,20,23] or four categories [29]. Sex-specific categories were also considered for alcohol consumption in some OBSs [26,27]. 

The higher the level of exposure to antioxidants, the higher the OBS, whereas the highest levels of pro-oxidant facts were associated with the lowest OBS. Therefore, higher OBS scores indicated beneficial oxidative balance and predominance of antioxidant components. This scoring scheme was applied in all OBS, except in the one developed by Van Hoydonck et al. [9]. Other noteworthy features are the adjustment for total energy intake in some OBSs [13,16,19,20,23,28] and the application of different weighting methods for scoring the components in others. For instance, there were some OBSs applying four weighting methods (e.g., equal, literature-based, *a posteriori*, and Bayesian, or effect measures for the association with FOP and FIP levels) [13,23,24], or two weighting methods (equal and based on effect measures with cancer risk) [12,30].

### 3.3. Rationale for the Inclusion of Antioxidant and Pro-Oxidant Components in OBSs 

There were both antioxidant and pro-oxidant components considered in OBSs. Some of the reasons that led to the consideration of these components in OBSs are outlined below.

The main mechanisms of antioxidants include free radical scavenging by single electrons or hydrogen atom transfer to reactive species; inhibition of expression, synthesis or activity of prooxidant enzymes involved in the formation of reactive species; the metal transition chelating effect of promoters of reactive species; and activation or induction of antioxidant enzymes. As for dietary antioxidants, the scores included Vitamin C, Vitamin E, carotenoids, flavonoids, and glucosinolates, among others. Vitamin C is a water-soluble antioxidant that scavenges oxygen-derived free radicals including those produced by lipid peroxidation [31]. This vitamin can also regenerate α-tocopherol, thereby contributing to its protection against lipid peroxidation [32]. Carotenoids have potential antioxidant properties because of their chemical structure (isoprenoids in chains of polyenes) and interaction with biological membranes. These molecules act as scavengers of ROS protecting against OS and are able to reverse OS-induced inflammation processes [33,34]. Among carotenoids, β and α-carotene, zeaxanthin β-cryptoxanthine and licopene are very efficient at neutralizing ROS by quenching singlet oxygen, neutralizing thyol radicals, and stabilizing peroxyl radicals [35,36]. Licopene is the carotenoid exerting the highest in vitro antioxidant activity. Its antioxidant potential consists in catching peroxide radicals and consequently inhibiting DNA damage and lipid peroxidation, including LDL lipoproteins [35,37,38]. Vitamin E comprises four tocopherols (α, β, γ, and δ) and four tocotrienols, with α-tocopherol being the most biologically active form. It is a fat-soluble compound with many physiologic functions against diseases initiated or promoted by oxygen radicals, by conferring protection against lipid peroxidation and by conserving the membrane integrity. Both β-carotene and α-tocopherol can act synergically in cell membranes to reduce lipid peroxidation [39]. Also, together with ascorbic acid, these compounds work synergistically against ROS and RNS. 

Flavonoids are compounds that belong to polyphenols, widely distributed in the plant kingdom. This group of compounds comprises several subclasses (e.g., flavones, flavonols, flavanones, flavanoles, anthocyanidins, and isoflavones) [40]. Some of its antioxidant functions are based on the donation of hydrogen to free radicals, the prevention of metal-catalyzing free-radical formation, and the integration of cell membranes to protect against lipid peroxidation [41,42]. More specifically, in vitro studies have shown that flavonoids inhibit enzymes involved in the generation of ROS, such as NADH oxidase, lipooxigenases and monooxigenases. Glucosinolates, secondary metabolites in Brassica vegetables, are sulfur compounds with antioxidant functions via their hydrolyzed forms (indoles and (iso)thiocyanates) [43]. Isothiocyanates have the ability to reduce the activation of procarcinogens by inhibiting phase 1 enzymes and inducing transcription of cytoprotective phase 2 enzymes. These functions involve the induction of electrophiles, the induction of hemoxygenase-1, which catalyzes heme to biliverdin, and the induction of glutathione peroxidase [44]. The induction of cytoprotective proteins could prevent chronic inflammation [45]. Within the Brassica vegetables, the crucifers were considered as an antioxidant food group in one of the OBSs [16]. This group contains glucosinolates but also folate, carotenoids, chlorophyll, and flavonoids.

Regarding minerals, Se is a cofactor of glutathione peroxidase (GPx), which reduces H_2_O_2_ including hydroperoxides generated by oxidation of fatty acids, phospholipids, and cholesterol. Glutathione reductase is also Se-dependent and responsible for maintaining the ratio GSH/GSSG [46]. Se is also a cofactor of thioredoxin reductase enzymes, which protect against oxidative damage by regenerating oxidized vitamin C [47]. Zn is another antioxidant mineral considered in OBSs. It is a cofactor of more than 300 enzymes and of more than 2000 transcriptional factors, of which some are involved in the endogenous antioxidant system. Prominent among these enzymes are superoxide dismutase (SOD) and GPx. This mineral also induces the synthesis of metallothioneins, through which the activity of ROS and NADPH-oxidase are blocked [48].

Other vitamins with antioxidant potential, though not considered by most of the OBSs, are folic acid and vitamin D. These vitamins have been shown to influence OS levels due to their antioxidative properties [49,50]. Folic acid has been reported to improve the endogenous antioxidant system. Also, folic acid increases the ratio of reduced/oxidized glutathione (GSH/GSSG) and reduces protein nitration [51]. The antioxidant potential of Vitamin D passes through the expression of the erythroid-derived 2 nuclear factor Nfr2, which under ROS production activates the expression of antioxidant enzymes. Vitamin D also increases the expression of the protein Klotho, which also regulates the expression of these enzymes [52]. 

Regarding dietary pro-oxidants, fats represent the component with the greatest pro-oxidant potential. Dietary fat intake by itself increases oxidative stress following lipid peroxidation. In particular, saturated fatty acids, palmitic acid, as well as stearic acid and myristic acid, increase ROS by this mechanism and trigger DNA damage since they also compromise the response to double-stranded breaks [53]. PUFAs also seem to increase the formation of radicals in cells through increased lipid peroxide formation. This type of fatty acid also regulates the genes responsible for transcription of antioxidant enzymes [54]. It is important to highlight that n-3 and n-6 PUFAs have a differing role in OS, either regulating or promoting OS in cells. Specifically, n-3 PUFAs (eicosapentaenoic acid (EPA, 20:5n-3) and docosahexaenoic acid (DHA, 20:6n-3)), although the mechanism of action are not fully understood, have been shown to be anti-inflammatory [55,56]. However, higher intakes of n-6 PUFAS (gamma-linolenic acid (GLA; 18:3n-6)) have been associated with an increased OS though increased free-radical production [57,58]. While OBSs included n-6 PUFAs on this basis, the mechanisms of action are also rather controversial. Among pro-oxidant minerals, iron intake (particularly heme iron) is one of the key components. Iron intake is associated with oxygen transport and storage in red cells. Owing to this, high iron levels could catalyze oxidative reactions, promoting iron-induced lipid and protein peroxidation [59,60]. Iron-amplified OS may also lead to DNA damage and oxidative activation of procarcinogens [61]. This mineral may also intensify OS by catalyzing the production of highly reactive hydroxyl radicals via the Haber‒Weiss reaction [62]. Likewise, red meat, the main dietary source of iron, could lead to OS through the same mechanisms [62]. Red meat was considered a pro-oxidant food group in one of the OBSs [16]. 

With respect to lifestyle factors, there were also antioxidant and pro-oxidant factors considered. The only antioxidant factor was physical activity, for which several studies have shown that, regardless of the intensity and type of exercise, physical activity tends to increase antioxidant markers, while pro-oxidant markers are decreased [63]. The mechanisms by which response to OS is activated are initiation of cellular antioxidant signaling systems and enhancement of the expression of antioxidant enzymes [64]. Alcohol intake and smoking are two of the main factors acting as pro-oxidants. It is generally recognized that alcohol consumption can increase ROS and subsequently inflammation. Chronic alcohol intake induces OS through oxidation of ethanol to acetaldehyde, which can lead to RONS production, nucleic acid oxidation, and decreased activity of antioxidant enzymes [65]. Smoking and alcohol intake together might more prominently affect OS redox balance. In fact, smoking is a fundamental factor in the assessment of the individual´s pro-oxidant status. Cigarette smoking has been shown to influence the levels of some antioxidants in plasma [66,67]. Tobacco is also related to inhalation of free radicals from smoke released during combustion [68,69], and smoking-induced free radicals have been detected in many tissues and organ systems [70]. The direct increase in the OS burden of inhaled tobacco smoke could be further enhanced in the lungs through the secondary release of oxygen radicals, which leads to an increase in blood/tissue markers of OS [67]. Another pro-oxidant factor included in most OBSs is obesity (body mass index, BMI, >30 kg/m^2^). OS induction in obesity has been related to metabolic switches and the involvement of redox-responsive signaling pathways in several clinical studies. These studies have elucidated not only a relationship between free radical biomarkers and BMI but also how several cell functions or tissues (including vascular endothelial cells, myocytes, or pancreatic-β-cells) are altered by OS-associated obesity, thereby leading to the development of metabolic diseases [71]. Experimental studies have also shown that adipose tissue enhances ROS production derived from lipid peroxidation and decreases antioxidant defense; the expression of antioxidant enzymes has been found to be lower in obese subjects [71].

Finally, medication components with a potential antioxidant potential, such as aspirin and non-steroidal anti-inflammatories (NSAID), have been also considered in OBSs. It has been reported that the use of aspirin inhibits the production of ROS in cells exposed to oxidized-LDL [72]. Together, aspirin and NSAIDs are involved in the regulation of ROS and RNS to prevent cellular damage and inflammation [72,73,74,75]. However, these functions have become controversial as recent studies also suggest that these drugs may increase the levels of oxidized proteins resulting from OS. For instance, NSAIDs have been shown to induce ROS in different cell types, including cardiac and cardiovascular-related cells [76].

Table 6 describes the rationale for considering some of the components (antioxidant and pro-oxidant factors) by virtue of the assessment of oxidative status, including dietary, biomarkers, medication components, and lifestyle factors. 

### 3.4. OBSs and Their Association with Health Outcomes

Table 7 shows a summary of the studies that have examined the association between OBSs and mortality, chronic diseases and health, or OS determinants. Some important aspects to be considered are that some components, mostly dietary components, are valued in quantiles (for the most part as tertiles), whereas others, mostly lifestyle factors and medication components, are considered with scoring based on pre-established categories. Another important aspect is the number of quantifiable divisions (cutoffs) and the contribution of each component to the overall score. These differences among the OBS are highlighted in the following sections.

#### 3.4.1. OBS and Mortality Risk

There were two studies on the association between adherence to an OBS and mortality. The OBS developed by Van Hoydonck et al., in 2002 was the first score [9] to assess whether individuals more prominent to oxidative imbalance were at greater risk of all-cause and cause-specific mortality. This OBS, based on three dietary components (Vitamin C as water-soluble vitamin, β-carotene as liposoluble vitamin, and iron), was developed within the Belgian Interuniversity Research on Nutrition and Health (BIRNH) study, which comprised 2814 male smokers aged 25–74 years. A higher OBS, reflecting a higher pro-oxidant exposure, was associated with a 44% (95% CI: 13%–82%) higher risk of all-cause mortality and 62% (7–145%) overall cancer mortality compared with men in the lowest score group irrespective of smoking duration or intensity, age, educational level, and total energy intake. This association was, however, not significant for cardiovascular disease (CVD) mortality risk (RR = 1.31; 95% CI: 0.86–2.00). Residual confounding is likely to have affected these findings since other OS-related factors were not accounted for (e.g., other antioxidant nutrients or physical activity). A second, much larger study, explored the association between a more complete OBS and mortality risk within the Reasons for Geographic and Racial Difference in stroke REGARDS study (*N* = 21,031 participants) [24]. The scoring methods used in this OBS were based on assigning to the components weights proportional to their contribution to oxidative balance, as either equal weights, literature-based weights, weights based on the magnitude of the association between each component and plasma/serum fluorescent oxidation products (FOP) levels, or weights based on the magnitude of the associations between each component and plasma/serum F2-isoprostanes (FIP) levels. 

The OBS based on equal weights resembled the OBS developed by Dash et al. [13], with some minor differences in its operationalization. This OBS score consisted of 14 components, including some dietary factors, lifestyle factors, and two medication components. For the equal weights OBS, every component was divided into tertiles that served to assign 0, one, or two points with increasing exposure to antioxidants. Weights for the components taken from literature research or from association studies between these components and OS-related biomarkers (FIP or FOP) were considered for the other OBSs. The multivariable adjusted HRs (95% CI) for all-cause, cancer and non-cancer mortality causes for those in the highest vs. the lowest equal-weighting OBS quartile were: 0.70 (95% CI: 0.61–0.81), 0.50 (95% CI: 0.37–0.67) and 0.77 (95% CI: 0.66–0.89), respectively (*p*-trend < 0.01). As in the study by Hoydonck et al., no association was found between the OBSs and CVD mortality, or for lung disease mortality. Interestingly, all associations were attenuated after removal of smoking from the OBSs, though statistically significant results remained for all-cause and cancer mortality. Similar results were observed across all OBSs.

#### 3.4.2. OBSs and Colorectal Adenoma Risk

A total of seven studies evaluated the association between adherence to an OBS and risk of colorectal adenomas. The OBS by Goodman et al. [10], the so-called Oxidative Stress Score (OSS), was first developed within two case-control studies on colorectal adenoma and prostate cancer: the adenomatous polyps MAP (Markers of Adenomatous Polyps) study and the MPC (Markers of Prostate Cancer) study, respectively. This OBSs presented 12 components with antioxidant components being diet or biomarker-based, some pro-oxidant components (e.g., saturated fat and iron intake or serum ferritin), lifestyle factors and medication factors. Subjects in the highest category of antioxidant components were given one point and those who were in the lowest level obtained 0 points; for antioxidant components the score was reversed. Within the MAP study, including 170 adenomas (histologically confirmed) and 230 adenoma free subjects, higher vs. low OSS values were associated with a reduced colorectal adenoma risk (OR = 0.45; 95% CI: 0.21–0.99). This OBS was updated and modified in another version [15] by assigning scores from 0 to 2, from high pro-oxidant to high antioxidant exposure, respectively. In this updated score, all dietary components were included as energy-adjusted variables [80] and both dietary and supplement intake of dietary factors was accounted for. After applying the score to 2305 subjects undergoing colonic endoscopy, it was found that a higher score as compared to the lowest score group (≤3 points) was associated with a reduced adenoma risk (OR for high vs. low OBS = 0.19). Three further modification were introduced to account for α- and β-carotene, α- and γ-tocopherol, and PUFAs, all of which were categorized based on tertiles. This OBS had an additional distinctive feature as it considered both questionnaire (PUFA and vitamin C) and biomarker-based dietary factors (e.g., α- and β-carotene). Another adaptation was the removal of alcohol consumption from this OBS. Within a smaller sample set of the MAP study, it was found that high vs. low scorings of this OBS were associated with a 34% reduced adenoma risk (95% CI: 0.13–0.88). For every additional point in the overall score, there was a reduction of 10 % (95% CI: 0.83–0.97) in adenoma occurrence. 

Dash et al. [13] applied different weighting methods to build the same OBSs, including two were *a priori*-derived methods (OBS-equal weight and OBS-literature review), an *a posteriori* method (OBS based on study data) and a score combining both *a priori* and data-driven methods (OBS-Bayesian). These OBS were developed within three case-control studies; the Cancer Prevention Research Unit (CPRU) study, and the MAP I and MAP II studies. All OBSs presented 15 components and included different carotenoids (α-carotene, β-carotene and β-cryptoxanthin) and other vitamins, some fatty acids (PUFAs n-3, PUFAs n-6 and SFA), flavonoids and glucosinolates, all being energy-adjusted by the residual method, and four nondietary components (smoking, alcohol, obesity and physical activity). Comparing the OR of the highest tertile vs. the lowest OBS, all four OBSs showed a statistically significant reduced adenoma risk with ORs ranging from 0.38 to 0.54, and a significant trend of association with increasing OBSs values. OBSs without the lifestyle components, i.e., the dietary form of the OBSs, were less prominently or not associated (OBS-lit review and OBS-Bayesian) with adenoma risk, whereas the lifestyle OBSs showed a stronger or similar reduced risk of colorectal adenoma than the complete OBSs. No differences in the association between the OBSs and adenoma risk were observed by colon and rectum site, but stronger associations were observed overall for advanced adenomas. 

Using again data from the CPRU and MAP-I/II studies (472 cases and 578 controls), Labadie et al. [20] developed another OBS made up of 11 components, similar to that of Goodman et al., 2008 but without Se supplements and incorporating SFA. Likewise, nutrient intakes including both dietary and supplemental intakes were energy-adjusted according to the residual method. The association between this OBS and colorectal adenoma risk was this time explored accounting for effect modification by the endogenous antioxidant network. A genetic score comprising genes encoding for SOD2 (eight SNPs), CAT (11 SNPs) and GSTP1 (five SNPs), following an additive model of inheritance (0, 1 and 2 points awarded for every high-risk allele) or a SNP—adenoma risk-specific score, was explored for this purpose. While this OBS was inversely associated with colorectal adenoma risk as in previous studies [10,15], no association was observed by the genetic score or individual variant genetic alleles.

Kong et al., applied another modified OBS [11] within the MAP-I and MAP-II studies. Unlike the previous OBSs [10,15,18], this OBS was characterized by the inclusion of several biomarker nutrient components except λ-tocopherol, and the same dietary components (vitamin C and PUFA intake), lifestyle components (smoking, alcohol, Se supplemental use) and medication components (NSAID and aspirin). High adherence to this OBS as compared to low adherence was associated with a 61% lower adenoma risk (95% CI: 0.17–0.89). A high OBS scoring was further associated with low levels of FIP, FOP, and CRP, confirming that this OBS is closely related to inflammation and OS. Finally, the OBS by Goodman et al. [10,15,18], including the modifications introduced by Dash et al. [13], was tested for its association with colorectal adenoma risk within the same study populations (CPRU, MAP I and MAP II studies) in the study by Wang et al. [28]. Effect modification by 16 genetic variants of base excision repair genotypes and their conjunction in a genetic risk score based on an additive genetic model of inheritance was explored in this association study. However, while a low OBS combined with high genetic score was associated with a higher adenoma risk, the interactions for OBS*gene regarding colorectal adenoma risk was not found to be statistically significant (*p* for interaction = 0.42). 

#### 3.4.3. OBSs and Colorectal Cancer Risk

Two studies have evaluated the association between adherence to an OBS and colorectal cancer risk. The association between an OBS [19] and colorectal cancer (CRC) risk, accounting for the interaction between diet and lifestyle factors and genes that modulate the impact of OS in the body, was evaluated within two study populations (KPMCP and Utah study) comprising 1555 colon cancer cases and 1956 controls, and 754 rectal cancer cases and 9959 controls, respectively. Unlike previous OBSs, this OBS included β-carotene rather than total carotenoids, vitamin D, calcium and folic acid, NSAID use and smoking status, while it discarded the consumption of alcohol. In addition, a polygenic score including genetic variants related to OS neutralization mechanisms (SNPs belonging to genes: OS2A, MPO, EPX and HIF1A), was built to explore its interaction with the OBS in relation to CRC risk (both an additive and recessive model of inheritance was considered). This OBS was associated with a reduced risk of both colon and rectal cancer (high vs. low OBS: OR = 0.52; 95% CI: 0.41–0.66 and OR = 0.49; 95% CI: 0.35–0.70, respectively). However, this association was modified by the genetic risk score in both colon (*p* for interaction: < 0.001) and rectal cancer (*p* for interaction: 0.002) in such a manner that a high-risk genotype and a low OBS conferred the highest colon (OR = 2.18; 95% CI: 1.36–3.50) and rectal (OR = 1.95; 95% CI: 1.02–3.75) cancer risk. Thus, this study showed that diet, lifestyle and genetic factors are associated with CRC risk potentially through OS, and that higher genetic susceptibility to OS further increases this risk. Furthermore, some SNPs of the NOS2A gene only showed interaction with dietary variables (calcium and folic acid) in rectal but not in colon cancer.

Dash et al., applied a similar OBSs to that published previously by the same authors [13,23], namely the four OBSs (equal weights, literature review-based, *a posteriori* data-based and weights based on Bayesian analysis) consisting of 15 components and including additionally supplemental selenium intake, to investigate the association between OBS and CRC risk among 1109 incident CRC cases that occurred within 80,063 subjects of the Cancer Prevention Study II (CPS-II). In this study, higher values of the four OBSs were associated with a 41–53% lower risk of CRC (RR high vs. low quartile): OBS-equal weight 0.59 (0.49–0.70), OBS-literature review 0.60 (0.50–0.73), OBS-*a posteriori* 0.47 (0.39–0.57) and OBS Bayesian 0.50 (0.41–0.61).

#### 3.4.4. OBS and Breast Cancer Risk

There was only one study on the association between adherence to an OBS and breast cancer risk. Slaterry et al., evaluated the interaction between a new mostly dietary-OBS and a large list of angiogenesis-related genetic variants in relation to breast cancer risk [21]. This OBS was applied to women belonging to the Breast Cancer Health Disparities study, a case-control study including 2111 Hispanic cases and 2597 controls, and 1481 cases non-Hispanic 1586 controls (aged 25–79 years). The majority were histologically confirmed invasive cancers, but some in-situ cancers (*n* = 341) were included as well. The OBS included five dietary components, all being antioxidants, and a single lifestyle and pro-oxidant factor (alcohol). A higher relative to low OBS was associated with a reduced breast cancer risk (OR = 0.74, 95% CI: 0.64–0.84) with this association being stronger for women of the highest Native American ancestry group (OR = 0.44, 95% CI: 0.30–0.65). Statistically significant associations were also found between the OBS components and breast cancer risk. Associations between the OBS or its components with ER/PR tumor subgroups could not be evaluated due to incomplete information. There were few significant interactions between the selected genetic variants and the OBS; only one (VEGFA rs3025033) remained significant after multiple test correction. Thus, the genes evaluated had a minor impact on the association between the OBS and breast cancer risk.

#### 3.4.5. OBSs and Prostate Cancer Risk

The association between adherence to an OBS and prostate cancer risk was examined in three studies. The OBS developed by Goodman et al., 2007, described above regarding the assessment of the OBS (i.e., OSS) and colorectal adenoma risk, was also applied to assess the association with prostate cancer risk within the MPC study including 112 prostate cancer cases and 258 controls. An inverse association between OSS and prostate cancer risk was found by comparing high vs. low OSS levels (OR = 0.28; 95% CI: 0.28–0.82), but no association was observed on the continuous scale per 10% increase in the OSS (OR = 0.90; 95% CI: 0.77–1.04). The corresponding ORs for high vs. low OSS levels within a subset of the same study population (97 cases and 226 controls) were 0.34 (95% CI: 0.14–0.86) when the modified version of the OSS (combining FFQ and biomarker dietary factors) was considered. 

The association between OBSs and prostate cancer risk was also evaluated using another comprehensive OBS [12], with component that were considered as either equal weights or literature-based weights established according to the reported association of these components with prostate cancer risk (pooled estimates). This score presented 15 dietary components including traditional components such as iron or vitamin C and other less commonly considered components such as flavonoids, glucosinolates, different type of fatty acids, and dietary zinc intake, and four lifestyle components and a medication component. Among 43,325 men participating in the Cancer Prevention Study II Nutrition Cohort and 3386 prostate cancer cases identified during follow-up, it was observed that those in the highest quartile of the OBSs relative to the lowest quartile had a higher risk of developing prostate cancer (HR for equal weights components = 1.17; 95% CI: 1.04–1.32; HR for literature-weight components = 1.15; 95% CI: 1.03–1.30). Higher prostate cancer risks were also observed for OBS assessed as a continuous variable. No statistically significant differences were observed by aggressiveness of the disease. Thus, this study contradicts findings of the Goodman´s OSS reporting an inverse association between OBS and prostate cancer. The two other cohort studies that evaluated the association between OBS and prostate cancer risk also found contradictory results. For instance, the OBS developed within a case-cohort study of 661 prostate cancer cases embedded in the Canadian Study of Diet, Lifestyle and Health cohort (CSDLH) study [16], which considered an OBS made up of eight dietary antioxidant components (e.g., cruciferous vegetables and some vitamins including Vitamins C and E carotenoids, and Se supplements) and five pro-oxidant dietary and lifestyle components (red, meat, iron, PUFAs, smoking and alcohol intake), with dietary components all adjusted for total energy intake [80], reported a null association between OBS and prostate cancer risk, overall (HR high vs. low OBS = 1.01; 95% CI: 0.74–1.36 and HR per 1 unit increment = 1.00; 95% CI: 0.99–1.01) or by cancer aggressiveness type. The authors also evaluated the association between each OBS component and prostate cancer risk confirming that there were in general null results. In the case-cohort study conducted in the Netherlands cohort study (NLCS study) including 3451 prostate cancer cases, Geybels et al. [17] developed an OBS that included five dietary components (vitamin C, vitamin E, carotenoids, and catechin intake as antioxidants and heme-iron as pro-oxidant) and two lifestyle components. This OBS was also not associated with prostate cancer risk (HR high vs. low OBS = 1.16; 95% CI: 0.98–1.36 and HR per unit increment = 1.01; 95% CI: 1.00–1.03). Null associations were also observed by stage of the disease (I/II, III/IV or IV). In an analysis by OBS components, only total catechin intake was inversely associated prostate cancer risk (HR high vs. low intakes = 0.76). While the association between OBS and prostate cancer seemed to be positive and significant in former smokers in the highest OBS category, it is uncertain whether effect modification by smoking status was statistically significant.

#### 3.4.6. OBS and Cardiovascular Disease (CVD) Risk Factors

There were four studies evaluating the association between adherence to an OBS and CDV risk factors. Among the CVD disease risk factors, some studies have explored the association between an OBS and hypertension, circulating lipids and the metabolic syndrome (MetS). Chronic kidney disease, likely leading to cardiovascular complications, has been also studied in relation to OBS.

As regards hypertension, data from 317 subjects participating in the Study of Race, Stress, and Hypertension (SRSH) study with blood pressure measures, and a OBS similar to the one developed by Lakkur et al. [12], but modified by removing other components (plasma levels of y-tocopherol) and including others (weight status), were considered. Similar to Lakkur et al., the scoring system was based on sex-specific tertiles, and the final OBS was categorized into three equal intervals. There was a statistically significant association between this OBS and hypertension in multivariate adjusted regression models (OR for high vs. low OBS = 0.17; 95% CI: 0.79–0.96 and OR per 1 unit increase in the score = 0.87; 95% CI: 0.79–0.96). No associations were observed between some OS-related markers (FIP, FOP and mtDNA) with systolic and diastolic blood pressure. 

Regarding lipid profiles and other markers of cardiovascular health, Lakkur et al. [26] developed another modified version of the OBS developed before by Dash et al. [13]. The modifications included a more the detailed assessment of carotenoids, the combination of PUFAs in the same component and the removal of flavonoids, glucosinolates, and saturated fats. Total dietary and supplemental intake was considered whenever possible. Adaptations on the lifestyle components included the inclusion of medication components (NSAID and aspirin) and the removal of physical activity and BMI. This OBS was applied to 19,825 subjects (aged ≥45 years) participating in the REGARDS cohort study. The ORs comparing highest vs. lowest OBS categories were, 0.50 (95% CI: 0.36–0.71) for waist circumference, and 0.75 (95% CI: 0.58–0.98) for LDL-cholesterol. The association between this OBS and HDL-cholesterol was statistically significant and inverse among females (OR = 0.48; 95% CI: 0.28–0.83), but positive among males (OR = 1.63; 95% CI: 1.09–2.45; *p* for interaction by sex < 0.01). No statistically significant associations were observed for serum albumin, total cholesterol and triglycerides.

The association between an OBS and the MetS was examined using the Korea Association Resource (KARE) data from 6417 participants [30]. This OBS only included seven components, of which four accounted for antioxidant exposure (intake of β-carotene, vitamin C, retinol, and physical activity) and three were pro-oxidant factors (smoking, alcohol, and iron intake). Of note is the fact that dietary data were not only self-reported but also based on a single 24-h recall. Higher scorings of this OBS compared to the lowest quantile was associated with significantly lower MetS risk regardless of the OBS weighting method (OR for equal weights = 0.65; 95% CI: 0.51–0.83, OR for beta-coefficients weights = 0.56; 95% CI: 0.76–0.41 and OR for principal components weights = 0.55; 95% CI: 0.40–0.75). These estimates remained similar when type 2 diabetes patients were excluded from the analyses. There was no association between this OBS with any of the MetS components except for waist circumference (β high vs. low OBS quantile = −0.98; *p*-value < 0.01). The authors conducted further a GWAS analysis to elucidate SNPs associated with the OBS also enriched in MetS biological processes. Interestingly, some of these SNPs belong to genes involved in angiogenesis, OS and inflammation.

The association between chronic kidney disease (CKD) and oxidative balance was examined for an OBS that was based on other previous ones [10,12,19] and adapted further by Llori et al. [27] by including total cryptoxanthin. Alcohol intake, NSAIDs, and aspirin were the only non-dietary components in this OBS. All dietary components were questionnaire-based and considered supplemental intakes. In this study, carried out within the REGARDS cohort study comprising 19,461 participants of which around 12% had albuminuria or CKD at baseline, higher OBS quartiles compared to the lowest quartile were associated with a 21% lower prevalence of CKD (95% CI: 0.67–0.92) and a 33% lower prevalence of macroalbuminuria (95% CI: 0.49–0.92). Significant associations were also observed per 5 or 10 units increase in the OBS with these two kidney disease markers (e.g., OR for CKD per 5 units OBS = 0.90; 95% CI: 0.84–0.97). The associations between OBS and albuminuria or incident end stage renal disease (90 events occurred during follow-up) were not statistically significant.

#### 3.4.7. OBS and Oxidative Stress and Inflammation Biomarkers 

Three studies explored how adherence to an OBS relates to biomarkers of inflammation and oxidative stress. In 2014, Lakkur et al., developed another OBS that comprised 13 components including measurements of antioxidant nutrients and serum, ferritin in plasma or serum samples (lycopene, α-carotene, β-carotene, β-cryptoxanthin, zeaxanthin, lutein, α-tocopherol and γ-tocopherol, ferritin), as well as lifestyle factors including physical activity, smoking and alcohol consumption, and medical components (NSAIDs and aspirin) [22]. This score was developed within the Study of Race, Stress, and Hypertension (SRSH) including 321 subjects (aged 25–74 years). The authors reported that there was an inverse association between the OBS and F2-isoprostanes (FIP; OR high vs. low OBS = 0.04; 95% CI: 0.01–0.17) indicating lower systemic OS, but an unexpected positive association with fluorescent oxidative products (FOP; OR high vs. low OBS = 5.64; 95% CI: 2.35–13.54). The results for mitochondrial DNA copy number (mtDNA) were unstable and analysis-dependent as risk estimates varied in study subjects with complete or imputed information. Interestingly, the three biomarkers were not inter-correlated, suggesting that non-oxidative products may affect levels of some of these biomarkers, most likely FOP. Using a modified version of this OBS [25], the OBS was also found to be negatively correlated with FIP (rho = −0.18) but positively correlated with FOP (rho = 0.3). Similar findings were reported by Kong et al., 2014 [11], whose OBS based on questionnaire and nutrient biomarkers was also found to be negatively associated with FIP (OR high vs. low OBS = 0.25; 95% CI: 0.10–0.65) but positively associated with FOP levels (OR high vs. low OBS = 3.48; 95% CI: 1.51–8.02). In this study, higher OBS scorings were also reported to be associated with lower levels of the inflammation marker C-reactive protein, CRP (OR high vs. low OBS = 0.21; 95% CI: 0.09–0.49). The OBS developed by Lakkur et al., 2015, also showed an inverse association with CRP (OR high vs. low OBS = 0.50; 95% CI: 0.38–0.66). Likewise, increasing levels of the OBS by Lee et al. [30], were also related to lower CRP levels (β for high vs. low OBS quantiles = −0.28; *p*-value = < 0.01). Another of the aforementioned studies that reported an association between an OBS and some OS biomarkers was the study by Dash et al. [13]. Some of the questionnaire-based OBS developed by these authors, the literature weight and the Bayesian-based OBSs, were associated with lower FIP plasma levels, with dietary OBS components being interestingly more strongly associated with this OS marker after adjustment for lifestyle OBS components, further validating the use of these OBSs as a surrogate for oxidative balance. 

Glutamyltransferase (GGT) levels as a biomarker of OS have been also considered as oxidative balance markers. The OBS considered for this purpose was developed within a Korean study population (the Korea National Health and Nutrition Examination survey study (KNHANES-V) of 2087 men and 2071 women (aged, 19–65 years) [29]. This OBS included fewer components than most of those described above. Notably, this OBS included total fat as a pro-oxidant component, but did not include vitamin E or medication components. The lifestyle factors considered were smoking, alcohol, BMI and physical activity, as in other previous OBSs. Based on sex-specific quartiles or defined cut points, 0, 1, 2 or 3 points were assigned with increasing exposure to antioxidant factors. The scoring was reversed for pro-oxidant factors. The multivariable adjusted OR 95% CI for high vs. low OBS levels in a relation to GGT were 0.05 (0.01–0.19) and 0.27 (0.09–0.78) for men and women, respectively (*p* for trend <0.01). Interestingly, only the associations between the lifestyle OBS components with GGT levels were statistically significant. However, the dietary information used as dietary OBS components relied on a single 24-h recall, which was likely insufficient to capture the whole dietary antioxidant and pro-oxidant uptake. Also, the nutrients considered were limited and probably not representative of the entire anti- and pro-oxidant nutrient intake.

## 4. Discussion

There is a great variety of OBSs in the literature, with different definitions of antioxidant and pro-oxidant components, and ways of scoring assignments. Several OBSs only contain minor adjustments of other previous ones, and are therefore almost equivalent in quantifying the antioxidant and pro-oxidant exposure. Others include not only different components but also differing scoring schemes. These issues hinder making comparisons across OBSs regarding their impact on health determinants and outcomes. Thus, while OBSs contemplate a holistic view of the exposure to antioxidant and pro-oxidant factors, their translation into health effects is still far from been established. However, overall, studies assessing the association between the OBS and health outcomes support that higher exposure to antioxidant factors reflected by higher OBS levels is associated with a lower all-cause and cancer-related mortality risk, as well as with a reduced colorectal adenoma risk. Some studies have also provided evidence for a potential beneficial effect of OBSs against the development of certain cancer types or cardiovascular disease risk factors. For other endpoints, such as prostate cancer, the association between OBSs and risk of developing this disease is still inconclusive. To date, no study has explored the impact of oxidative balance by means of an OBSs on neurodegenerative diseases associated with the aging process.

The main purpose of the *a priori* OBS is to assess exposure to anti- and pro-oxidant components, considering the combination of dietary and biomarkers factors (nutrients, non-nutritive components and foods), lifestyle and medication factors [10]. This tool is essential to evaluate oxidative status in epidemiological studies in a relatively straightforward way, linking OS balance to disease risk. For instance, as reported in the study by Goodman et al., 2007 [10], when components of the OBS were individually tested for their association with colorectal adenomas or prostate cancer risk, different associations, often in opposite directions, were observed. Conversely, consistent associations for both outcomes were observed when the OBS was considered. Other studies also failed to show an association between specific OBSs components and the main outcomes [10,11,12,15,17,18,24,25,26,29,30] supporting that combined measures of antioxidant and pro-oxidant components may be associated to a greater extent with disease outcomes than the simple sum of anti- and pro-oxidant components [27]. The role of inflammation as both a cause and a result of OS is supported by a considerable body of evidence. Some OBSs, in turn, showed statistically significant associations with OS biomarkers (e.g., FIP) and/or inflammation markers (e.g., CRP), proving further the validity of the OBS for oxidative balance assessment [11,13,22,25,26,30].

Despite OS-related diseases being a major public health concern, known to be caused by the deleterious effects of free radicals on human cells, there are still some gaps in our knowledge concerning the exact mechanism by which antioxidants and pro-oxidants exert their effect on disease risk. In the present review, the antioxidant or pro-oxidant mechanisms of every OBS component have been revised to warrant their inclusion in the scores. There are, however, interactions between these components that may result in much more complex mechanisms underlying the OS and inflammation response in disease. This leads to a potential constraint when selecting the antioxidant and pro-oxidant components in the OBSs, as they could be correlated and mutually interacting, affecting the associations between the OBS and disease risks. This issue was taken into consideration in two of the OBS studies [16,19] by considering different weighting approaches for developing the OBS. Individual weights of the components of these OBSs featured were selected upon the research accumulated in relation to risk factors and diseases. By contrast, most OBSs considered that all antioxidant and pro-oxidant components likely exert the same effect on the oxidative balance of the body (equal-weights OBS). In addition to the above mentioned issue, the latter may be less adequate and robust given that every component is known to have a different antioxidant or pro-oxidant power. In fact, the redox potential of each antioxidant vitamin is variable. Thus, the biological contribution of each component to the oxidative status could be different [11]. For instance, lycopene may be a more prominent antioxidant than other carotenoids. Nevertheless, while weighted OBSs should be better suited to assess the balance of anti- and pro-oxidant factors, there were no major differences when compared with the unweighted OBS with regard to the association with risk of colorectal adenoma and other endpoints [12,13,24,30]. Thus, both ways of building OBSs can be appropriate. Also, *a posteriori* techniques have been used for the development of OBSs in epidemiological studies. Their drawback is, however, the limited translational capacity of these OBS into public health interventions. Therefore, the few publications of *a posteriori* OBSs have not been included in this review [13,14,23]. Other methodological approaches, including statistical methods accounting for the nature of OBS variables (e.g., correlation) or methods to encompass the complexity of the phenomenon, have not been considered. It is important to emphasize that *a priori* derived OBSs are ideal in epidemiological studies for establishing the effect of components related to OS, as a whole and individually, on health outcomes. Intervention studies proving these effects, by assessing the impact of a multiple-component intervention on health, will give extra knowledge and a solid foundation for health protection by modulating the individual´s oxidative balance.

Numerous differences between the OBSs were noted. Among the nutrient antioxidants, the most commonly used were vitamin C, vitamin E and some carotenoids, as they are key dietary antioxidants. However, not all OBSs included a complete set of these antioxidants due to a lack of data, specifically some carotenoids [9,21,29,30] Flavonoids and some polyphenols were seldom included [12,13,17,28] as well as folic acid [19,21], minerals such as zinc [12] or foods such as cruciferous vegetables or red meat [16]. The inclusion of food groups, indeed, was infrequent in this type of scores. Few OBS also considered Se in the OBS, which may lead to inappropriate oxidative balance assessment as questionnaire-based Se is known to be inaccurate [10]. Other ways of measuring the global antioxidant exposure in the diet, such as non-enzymatic antioxidant capacity (NEAC) [81], have been not considered yet. Another important aspect of the dietary antioxidant components of the scores is the inclusion of dietary supplements to account for the total intake of antioxidant nutrients. This was considered in nine of the OBSs [11,13,15,16,20,23,26,27,28] but the contribution of antioxidants from supplements might be negligible given their low use in the population and their weak association with chronic diseases when considered independent of dietary antioxidant intake [82].

Several OBSs lacked information on endogenous factors that modify OS such as genes encoding for antioxidant enzymes (the body’s endogenous antioxidant system) or base excision repair genes. This is probably one of the most important limitations that the OBSs presented. Nevertheless, the contribution of the individual’s genotype variation to the OS balance is difficult to assess as little is known on this issue [19]. In addition, those OBSs accounting for genetic variants of these genes did not show significant interactions with the OBS in relation to disease risk [19,20,21,28]. However, while these intrinsic factors did not seem to modify the effect of the OBSs on disease risk, more studies are warranted to confirm this lack of interaction. Other factors such as the gut microbiota composition may also have a role in the modulation effects of antioxidant and pro-oxidant factors. Differences were also noted in the way the components were valued as either anti- or pro-oxidant components. For example, total PUFAs were considered as pro-oxidant factors [15,16,19,24,26,27] unless a distinction between omega-3 (antioxidant components) and omega-6 PUFAs (pro-oxidant components) was made [12,13,23]. These changes on the consideration of a component being either anti- or pro-oxidant is because their role is much more complex than previously thought [83]. Another important aspect that could explain the different results of the studies is that in some studies dietary components of the OBS were adjusted for total energy intake [16], whereas other studies did not [10]. Also, the majority of the OBSs considered questionnaire-based dietary data with only five OBSs [10,11,18,22,25] considering biomarker-based dietary components. The latter are more reliable as they account for the real pool of dietary antioxidants and pro-oxidant factors in the body. However, these biomarkers are likely to reflect more short-term antioxidant/pro-oxidant effects. Among the questionnaire-based OBSs, FFQs were the most commonly used dietary assessment methods, despite these questionnaires present several limitations regarding the quantification of nutrients antioxidant intake during the different seasons [84]. Interestingly, the study by Kong et al., showed similar results when the OBSs components were derived from FFQs or by replacing the questionnaire information by real (biomarker-based) antioxidant and pro-oxidant exposures [11], proving that the questionnaire data are equally valid to derive optimal OBSs.

## 5. Conclusions

This review provides an overview of OBSs published in the literature regarding the components included, their definition in the score, and their effects on different health outcomes. The use of OBSs to account for the combined effect of antioxidants and pro-oxidants, considering potential synergies and antagonism among foods, nutrients, lifestyle factors, medications, and enzymatic genetic variants, is a valuable approach to understand the relationship of certain OS-related aspects of diet, lifestyle factors, and disease from an epidemiological point of view. Although the components most commonly included in the OBSs are very similar, there are also many differences among them. While this tool assesses the oxidative balance of an individual in a relatively simple way, more universal antioxidant components could be considered to cover the complexity of the antioxidant and pro-oxidant network. Unified methodological criteria for the definition of OBSs are also required to allow for comparisons between the studies assessing the association between an individual’s OB and a certain health outcome.

## Figures and Tables

**Table 1:** Description of the *a priori* Oxidative Balance Scores (OBSs), components, and scoring systems.

Author (s), Year	*N* Components	OBSs Components	Type of Components	Scoring per Component	Score Range
Van Hoydonck et al., 2002 [9]	3	2 Antioxidant/ 1 Pro-oxidant	Dietary	1–3	3–9
Goodman et al., 2007 [10]	12	9 Antioxidant/ 3 Pro-oxidant	Dietary, biomarkers, lifestyle and medication	0–1	0–12
Goodman et al., 2008 [15]	12	8 Antioxidant/ 5 Pro-oxidant	Dietary, lifestyle and medication	0–2	0–24
Goodman et al., 2010 [18]	14	11 Antioxidant/ 3 Pro-oxidant	Dietary, biomarkers, lifestyle and medication	0–2	0–28
Agalliu et al., 2011 [16]	13	8 Antioxidant/ 5 Pro-oxidant	Dietary and lifestyle	0–4	0–52
Slattery et al., 2012 [19]	13	10 Antioxidant/ 3 Pro-oxidant	Dietary, lifestyle and medication	0–2	0–26
Geybels et al., 2012 [17]	8	5 Antioxidant/ 3 Pro-oxidant	Dietary and lifestyle	0–3	0–24
Dash et al., 2013 [13]	15	9 Antioxidant/ 6 Pro-oxidant	Dietary and lifestyle	−1–1	−6–9
Labadie et al., 2013 [20]	11	7 Antioxidant/ 4 Pro-oxidant	Dietary, lifestyle and medication	0–2	0–22
Kong et al., 2014 [11]	14	10 Antioxidant/ 4 Pro-oxidant	Dietary, biomarkers, lifestyle and medication	0–2	0–28
Slattery et al., 2014 [21]	6	5 Antioxidant/ 1 Pro-oxidant	Dietary	0–2	0–12
Lakkur et al., 2014a [12]	20	14 Antioxidant/ 6 Pro-oxidant	Dietary, lifestyle and medication	0–3	0–60
Lakkur et al., 2014b [22]	13	10 Antioxidant/ 3 Pro-oxidant	Dietary, biomarkers, lifestyle and medication	0–2	0–26
Dash et al., 2015 [23]	16	10 Antioxidant/ 6 Pro-oxidant	Dietary and lifestyle	−1–1	−6–10
Kong et al., 2015 [24]	14	10 Antioxidant/ 4 Pro-oxidant	Dietary, lifestyle and medication	0–2	0–28
Annor et al., 2015 [25]	13	9 Antioxidant/ 4 Pro-oxidant	Dietary, biomarkers, lifestyle and medication	0–2	0–26
Lakkur et al., 2015 [26]	14	10 Antioxidant/ 4 Pro-oxidant	Dietary, lifestyle and medication	0–2	0-–28
Ilori et al., 2015 [27]	13	10 Antioxidant/ 3 Pro-oxidant	Dietary and medication	0–2	0–26
Wang et al., 2017 [28]	15	9 Antioxidant/ 6 Pro-oxidant	Dietary and lifestyle	0–2	0–30
Cho et al., 2017 [29]	8	3 Antioxidant/ 5 Pro-oxidant	Dietary and lifestyle	0–3	0–24
Lee et al., 2017 [30]	7	4 Antioxidant/ 3 Pro-oxidant	Dietary and lifestyle	0–2	0–14

**Table 2:** Lifestyle factors and medication components included in each *a priori* Oxidative Balance Score.

Author (s), Year	Lifestyle Factors Components			Medication Components	
	Antioxidant	Pro-Oxidant		Antioxidant	
	Physical Activity	Smoking History	BMI	Aspirin	Other NSAID
Van Hoydonck et al., 2002 [9]					
Goodman et al., 2007 [10]		X		X	X
Goodman et al., 2008 [15]		X		X	X
Goodman et al., 2010 [18]		X		X	X
Agalliu et al., 2011 [16]		X			
Slattery et al., 2012 [19]		X			X
Geybels et al., 2012 [17]		X			
Dash et al., 2013 [13]	X	X	X		
Labadie et al., 2013 [20]		X		X	X
Kong et al., 2014 [11]		X		X	X
Slattery et al., 2014 [21]					
Lakkur et al., 2014a [12]	X	X	X		X
Lakkur et al., 2014b [22]	X	X		X	X
Dash et al., 2015 [23]	X	X	X		
Kong et al., 2015 [24]		X		X	X
Annor et al., 2015 [25]	X	X	X	X	X
Lakkur et al., 2015 [26]		X		X	X
Ilori et al., 2015 [27]				X	X
Wang et al., 2017 [28]	X	X	X ^a^		
Cho et al., 2017 [29]	X	X	X		
Lee et al., 2017 [30]	X	X			

BMI: Body Mass Index; NSAID: Non-Steroidal Anti-Inflammatory Drug. ^a^ Obesity and waist: hip ratio.

**Table 3:** Dietary components included in each *a priori* Oxidative Balance Score.

Author (s), Year	Dietary Antioxidants ^a^																Dietary Pro-Oxidants ^a^				
	C	B_9_	β-car	Lyco	β-cryp	Lute/Zeaxan	Retinol	D	E	Se	Zn	Ca	Fiber	Flav	GCS	Catechin	Fat	PUFAS	SFA	Fe	Alcohol
Van Hoydonck et al., 2002 [9]	X		X																	X	
Goodman et al., 2007 [10]	X		X ^b^	X	X	X ^c^			X ^d^	X ^e^									X	X ^f^	
Goodman et al., 2008 [15]	X ^f^		X ^f,g^	X		X			X ^f^	X ^e^								X		X ^f^	X
Goodman et al., 2010 [18]	X									X ^e^								X			
Agalliu et al., 2011 [16]	X ^f^		X ^f^	X	X	X ^c^			X ^f^	X ^e^								X		X ^f^	X
Slattery et al., 2012 [19]	X	X	X	X		X		X	X	X		X						X		X	
Geybels et al., 2012 [17]	X		X	X					X							X				X ^n^	X
Dash et al., 2013 ^f^ [13]	X		X ^g^	X	X	X			X					X	X			X ^h^	X	X	X
Labadie et al., 2013 [20]	X ^f^		X ^f,i^	X ^f^		X ^f^			X ^f^										X	X	X
Kong et al., 2014 [11]	X ^f^									X ^e^								X			X
Slattery et al., 2014 [21]	X	X	X						X				X								X
Lakkur et al., 2014a [12]	X ^f^		X ^j^	X	X	X			X ^f^	X ^f^	X			X	X			X	X	X ^f^	X
Lakkur et al., 2014b [22]																					X
Dash et al., 2015 ^f^ [23]	X		X ^g^	X	X	X			X	X ^e^				X	X			X ^h^	X	X	X
Kong et al., 2015 [24]	X ^f^		X ^f,j^	X ^f^	x ^f^	X ^f^			X ^f,k^	X								X		X ^f^	X
Annor et al., 2015 [25]																					X
Lakkur et al., 2015 ^f^ [26]	X ^f^		X _j_	X	X	X			X ^f^	X ^f^								X	X		X
Ilori et al., 2015 [27]	X ^f^		X ^j^	X	X ^l^	X			X ^f^	X ^e^								X		X ^f^	X
Wang et al., 2017 ^f^ [28]	X ^f^		X ^g^	X		X ^m^			X ^f^					X	X			X ^h^	X	X ^f^	X
Cho et al., 2017 [29]	X		X														X			X	X
Lee et al., 2017 [30]	X		X ^g^				X													X	X

Dietary components: C: vitamin C; β-car: β-carotene; lyco: lycopene; β-cryp: β-cryptoxanthin; lute/zeaxan: lutein/zeaxanthin; D: vitamin D; E: vitamin E; flav: flavonoids; GCS: glucosinolates; PUFAS: polyunsaturated fatty acids; SFA: Saturated Fatty Acids. ^a^ Questionnaire-based (Food Frequency Questionnaire) or 24-h recall dietary components considered. ^b^ α and β carotene intake for MAP study and plasma β carotene for MPC study. ^c^ Lutein/zeaxanthin for MAP study and only lutein intake for MPC study. ^d^ Total (α, β, γ and δ) tocopherol intake for MAP study and plasma α-tocopherol for MPC study. ^e^ Supplemental intakes. ^f^ Total intake = dietary intake and supplemental intake. ^g^ Includes total intake of plant-derived pro-vitamin A carotenes. ^h^ PUFAS-6 (pro-oxidant components) and PUFAS-3 (antioxidant components) as separate components. Lakkur et al., 2014a, omega-3 fatty acids. ^i^ Specifies the inclusion of carotenoids. ^j^ Includes α and β- carotene (dietary and supplemental intake) as separate components. ^k^ Specifies only α-tocopherols. ^l^ Total cryptoxanthin. ^m^ Only included lutein intake. ^n^ Heme iron intake.

**Table 4:** Biomarkers and food components included in each *a priori* Oxidative Balance Score.

Author (s), Year	Biomarker Components ^a^									Food Components	
	Antioxidant								Pro-Oxidant	Antioxidant	Pro-Oxidant
	α-carotene	β-carotene	Lycopene	Cryptoxanthin	Zeaxanthin	Lutein	α-tocopherol	γ-tocopherol	Ferritin	Crucifers	Red Meat
Van Hoydonck et al., 2002 [9]											
Goodman et al., 2007 [10]		X ^b^					X ^c^				
Goodman et al., 2008 [15]											
Goodman et al., 2010 [18]	X	X	X	X ^d^	X ^e^	X	X	X	X ^f^		
Agalliu et al., 2011 [16]										X	X
Slattery et al., 2012 [19]											
Geybels et al., 2012 [17]											
Dash et al., 2013 [13]											
Labadie et al., 2013 [20]											
Kong et al., 2014 [11]	X	X	X	X ^d^		X	X		X		
Slattery et al., 2014 [21]											
Lakkur et al., 2014a [12]											
Lakkur et al., 2014b [22]	X	X	X	X	X		X	X	X		
Dash et al., 2015 [23]											
Kong et al., 2015 [24]											
Annor et al., 2015 [25]	X	X	X	X	X		X		X		
Lakkur et al., 2015 [26]											
Ilori et al., 2015 [27]											
Wang et al., 2017 [28]											
Cho et al., 2017 [29]											
Lee et al., 2017 [30]											

^a^ Plasma- or serum-derived measurement. ^b^ Plasma β-carotene only was included in Oxidative Stress Score for prostate cancer in MPC study. ^c^ Plasma α-tocopherol only was included in Oxidative Stress Score for prostate cancer in MPC study. ^d^ Only included β-cryptoxanthin. ^e^ Zeaxanthin and lutein included in the same component. ^f^ For MPC study serum samples were no available and urinary selenium were available for MAP study.

**Table 5:** Methodological criteria of the Oxidative Balance Scores.

Author (s), Year	Cut-off Values		Scoring System for Each Component	Overall Score	Energy Adjustment and Other Methodological Issues
Van Hoydonck et al., 2002 [9]	Population-dependent	3 population-dependent dietary components (based on tertiles of intake).	The intakes were scored from 1 to 3 for pro-oxidant factors and from 3 to 1 for antioxidant factors. High score group (a diet poor in antioxidant and rich in iron).	The overall score ranged between 3 and 9 points. The score was divided into three groups: low (score 3–5), intermediate (score 6) and highest group (score 7–9).	Questionnaire-based (24-h recall) dietary components were considered. Energy adjustment was not considered.
Goodman et al., 2007 [10]	Predefined and population-dependent	8 population-dependent dietary/biomarker components (based on median intakes); four predefined components for smoking (never, ever), Se supplements (yes, no), and medication (NSAID or aspirin use, non-use).	All dietary/biomarker components were divided into dichotomous categories based on the median value. For antioxidants, one point was awarded for high-level exposure and 0 for low-level exposure. The score was reversed for pro-oxidant components. More points were awarded to antioxidant categorical variables (e.g., non-smokers, and NSAID users), and fewer points to pro-oxidant categories.	The overall score ranged between 0 and 24 points. The score was divided into three groups: low (score ≤2), intermediate (score 3–6) and highest antioxidant group (score ≥7).	Questionnaire and biomarker-based dietary components were considered. Energy adjustment was not considered.
Goodman et al., 2008 [15]	Predefined and population-dependent	7 population-dependent dietary components (based on sex-specific tertiles); five predefined components for smoking (never, former, current), Se supplements (yes, no), alcohol intake (low, moderate, heavy), and medication (NSAID or aspirin use, non-use).	All dietary components were divided into three categories based on the tertile values. For antioxidants, two points were awarded for high-level exposure, one point for intermediate, and 0 for low-level exposure. The score was reversed for pro-oxidant components. More points were awarded to antioxidant categorical variables (e.g., non-smokers and NSAID users), and conversely; fewer points to pro-oxidant categories.	The overall score ranged between 0 and 24 points. The OBSs was considered an ordinal variable.	Questionnaire-based (FFQ) dietary components were considered. Dietary components were adjusted for total energy intake.
Goodman et al., 2010 [18]	Predefined and population-dependent	10 population-dependent dietary/biomarker components (based on tertiles) and four predefined components for smoking (never, former, current), Se supplements (yes, no), and medication (NSAID or aspirin use, non-use).	All dietary/biomarker components divided into three categories based on the tertile values. For antioxidants, two points were awarded for high-level exposure, one point for intermediate, and 0 for low-level exposure. The score was reversed for pro-oxidant components. More points were awarded to antioxidant categorical variables (e.g., non-smokers, and NSAID users), and fewer points to pro-oxidant categories.	The overall score ranged between 0 and 24 points. The score was divided into three equal intervals: low (score 1–7), intermediate score (8–14), and highest antioxidant group (score 15–22).	Questionnaire and biomarker-based dietary components were considered. Energy adjustment was not considered.
Agalliu et al., 2011 [16]	Population-dependent	11 population-dependent dietary components (based on quintiles) and two population-dependent lifestyle components for smoking in pack-years and alcohol intake (in quartiles).	All dietary components were divided into five categories based on the quintile values. For antioxidants, four points were awarded for high-level exposure, one to three point for intermediate levels, and 0 for low-level exposure. The score was reversed for pro-oxidant components.	The overall score ranged between 0 and 52 points. The score was divided into five equal intervals.	Questionnaire-based (FFQ) dietary components were considered. All nutrients were energy-adjusted. Nutrient values included dietary and supplemental sources.
Slattery et al., 2012 [19]	Predefined and population-dependent	11 population-dependent dietary components (three categories for every component) and two predefined components for smoking (never, current smokers) and medication (NSAID use: never or recent/current use).	All dietary components were divided into three categories based on the tertile values. For antioxidants, two points were awarded for high-level exposure, one point for intermediate, and 0 for low-level exposure. The score was reversed for pro-oxidant components. More points were awarded to antioxidant categorical variables (e.g., non-smokers, and NSAID users), and fewer points to pro-oxidant categories.	The overall score ranged between 0 and 26 points. The score was divided into four groups: high risk (3–10), intermediate (11–13 and 14–16) and low risk (17–23).	Questionnaire-based (FFQ) dietary components were considered. Energy adjustment was considered in analyses evaluating the interaction between the polygenic score and the dietary variables.
Geybels et al., 2012 [17]	Predefined and population-dependent	5 population-dependent dietary components (based on quartiles for every component) and two predefined components for smoking (never, current smokers) and alcohol (abstainers, and predefined levels of intake).	All dietary components were divided into four categories based on the quartile values. For antioxidants, two points were awarded for high-level exposure, one point for intermediate, and 0 for low-level exposure. The score was reversed for pro-oxidant components. More points were awarded to antioxidant categorical variables (e.g., non-smokers, and alcohol abstainers), and fewer points to pro-oxidant categories.	The overall score ranged between 0 and 26 points. The score was divided into four groups: low (score 4), intermediate (score 7 and 9), and highest antioxidant group (score 12).	Questionnaire-based (FFQ) dietary components were considered. Energy adjustment was not considered.
Dash et al., 2013 [13]	Predefined and population-dependent	11 population-dependent dietary components (two categories for every component) and four non-dietary components for smoking, alcohol intake, obesity and physical activity.	Four methods were used of weighting all components: OBS-equal weight, OBS-lit review, OBS-*a posteriori* and OBS-Bayesian. For OBS-equal weight, all components were transformed to a standard normal distribution and multiplied by weights considered as +1 for antioxidants and −1 for pro-oxidants. For the other OBS, weights were calculated based on reported risk estimates, risks estimated within the study population or Bayesian analysis.	Transformed variables were multiplied by their weights and summed to generate the overall OBS. The scores were divided into tertiles: low, intermediate, and highest antioxidant group.	Questionnaire-based (FFQ) dietary components were considered. All nutrients were energy-adjusted. Nutrient values included dietary and supplemental sources.
Labadie et al., 2013 [20]	Predefined and population-dependent	7 population-dependent dietary components (based on sex-specific tertiles) and four predefined components for smoking (never, former, current), alcohol intake (low, moderate and heavy), and medication (NSAID or aspirin use, non-use).	All dietary components were divided into three categories based on the tertile values. For antioxidants, two points were awarded for high-level exposure, one point for intermediate, and 0 for low-level exposure. The score was reversed for pro-oxidant components. More points were awarded to antioxidant categorical variables (e.g., non-smokers, and NSAID users), and fewer points to pro-oxidant categories.	The overall score ranged between 0 and 22 points. The score was divided into two categories (high vs. low).	Questionnaire-based dietary components were considered. Dietary components were adjusted for total energy intake.
Kong et al., 2014 [11]	Predefined and population-dependent	9 population-dependent dietary/biomarkers components (tertiles for every component) and five predefined components for smoking (never, former, current), Se supplements (yes, no), alcohol intake (low, moderate, heavy), and medication (NSAID or aspirin use, non-use).	All dietary/biomarker components were divided into three categories based on the tertile values. For antioxidants, two points were awarded for high-level exposure, one point for intermediate, and 0 for low-level exposure. The score was reversed for pro-oxidant components. More points were awarded to antioxidant categorical variables (e.g., non-smokers, and NSAID users), and fewer points to pro-oxidant categories.	The overall score ranged between 0 and 28 points. The score was divided into three equal intervals: low (score 2–9), intermediate (score 10–16), and highest antioxidant group (score 17–24).	Questionnaire and biomarker-based dietary components were considered. Energy adjustment was not considered.
Slattery et al., 2014 [21]	Population-dependent	6 population-dependent dietary components (quartiles for every component) including alcohol.	All dietary components were divided into four categories based on the quartile values. For antioxidants, two points were awarded for high-level exposure (4th quartile), one point for intermediate levels, and 0 for low-level exposure (1st quartile). The score was reversed for pro-oxidant components.	The overall score ranged between 0 and 12 points. The score was divided into quartiles: low, intermediate and high antioxidant group.	Questionnaire-based (FFQ) dietary components were considered. Energy adjustment of nutrients per 1000 calories.
Lakkur et al., 2014a [12]	Predefined and population-dependent	15 population-dependent dietary components (quartiles); five predefined components for smoking (never, former, current), alcohol, BMI (normal, overweight, obese), physical activity and medication (NSAID use, non-use).	All dietary components were divided into four categories based on the quartile values. For antioxidants, two points were awarded for high-level exposure (4th quartile), one point for intermediate levels, and 0 for low-level exposure (1st quartile). The score was reversed for pro-oxidant components. Two weighting methods were applied: equal weights and literature-based weights.	The overall score ranged between 0 and 60 points. The score was divided into tertiles intervals or quartiles: low (scores 4–11, 5–10 and 4–12), intermediate (score 12–14, 11–15 and 13–15), and highest antioxidant group (score 15–22, 16–21 and 16–23).	Questionnaire-based (FFQ) dietary components were considered. Energy adjustment was not considered.
Lakkur et al., 2014b [22]	Predefined and population-dependent	8 population-dependent dietary/biomarkers components (tertiles) and one population-dependent lifestyle factors (physical activity in tertiles) and four predefined components for smoking (non-smokers and smokers), alcohol intake (non-drinkers and drinkers), and aspirin or NSAID medication (use, non-use).	All dietary/biomarker components were divided into three tertile values. For antioxidant components: two points were awarded for high-level exposure, one point for intermediate, and 0 for low predominance of antioxidants. The score was reversed for pro-oxidant components. More points were awarded to antioxidant categorical variables (non-smokers, non-drinkers and NSAID use), and fewer points to pro-oxidant categories.	The overall score ranged between 0 and 23 points. The score was divided into three groups based on tertiles.	Biomarker-based and dietary (FFQ) components were considered.
Dash et al., 2015 [23]	Predefined and population-dependent	11 population-dependent dietary components (two categories for every component) and four non-dietary components for smoking, alcohol intake, obesity and physical activity.	Four methods were used of weighting all components: OBS-equal weight, OBS-lit review, OBS-*a posteriori* and OBS-Bayesian For OBS-equal weight, all components were multiplied by their weights considered as +1 for antioxidants and −1 for pro-oxidants. For the other OBS, these weights were calculated based on reported risk estimates derived from reviews/ meta-analysis or study data or Bayesian analysis.	All components were multiplied by their weights and summed to generate the overall OBS. The scores were divided into quartiles.	Questionnaire-based (FFQ) dietary components were considered. All nutrients were energy-adjusted. Nutrient values included dietary and supplemental sources, except selenium intake (only supplemental intake).
Kong et al., 2015 [24]	Predefined and population-dependent	10 population-dependent dietary components (sex-specific tertiles for every component) and four predefined components for smoking (never, former, current), alcohol consumption (non-drinkers, moderate and heavy drinkers), and NSAID medication (use, non-use).	All dietary components were divided into three tertile values. For antioxidant components: two points were awarded for high-level exposure, one point for intermediate, and 0 for low predominance of antioxidants. The score was reversed for pro-oxidant components. More points were awarded to antioxidant categorical variables (non-smokers, non-drinkers and NSAID use), and fewer points to pro-oxidant categories. Four weighting methods were applied: equal weights, literature-based weights, weights based on the association with biomarkers levels	The overall OBS ranged between 0 and 28 points. The score was divided into four groups based on quartiles.	Questionnaire-based (FFQ) dietary components were considered.
Annor et al., 2015 [25]	Predefined and population-dependent	7 population-dependent dietary/biomarkers components (tertiles); one population-dependent lifestyle factors (physical activity tertiles) and five predefined components for smoking (non-smokers, current smokers), alcohol intake (non-drinkers, drinkers), and aspirin or NSAID medication (use, non-use), and BMI (normal, overweight and obese).	All dietary/biomarker components were divided into three tertile values. For antioxidant components: two points were awarded for high-level exposure, one point for intermediate, and 0 for low predominance of antioxidants. The score was reversed for pro-oxidant components. More points were awarded to antioxidant categorical variables (non-smokers, non-drinkers, normal weight, and NSAID use), and fewer points to pro-oxidant categories.	The overall score ranged between 0 and 26 points. The score was divided into three groups based on tertiles.	Biomarker-based and dietary components were considered.
Lakkur et al., 2015 [26]	Predefined and population-dependent	10 population-dependent dietary components (tertiles) and four predefined components for smoking (non-smokers and current smokers), alcohol intake (non-drinkers, moderate, heavier drinkers), and aspirin or NSAID medication (use, non-use).	All dietary components were divided into three tertile values. For antioxidant components: two points for high-level exposure, one point for intermediate, and 0 for low predominance of antioxidants. The score was reversed for pro-oxidant components. More points were awarded to antioxidant categorical variables (non-smokers, non-drinkers and NSAID use), and fewer points to pro-oxidant categories.	The overall score ranged between 0 and 28 points. The score was divided into five equal groups: score 3–7, score 8–12, score 13–17, score 18–21 and score 22–26.	Questionnaire-based (FFQ) dietary components were considered. Energy adjustment was not considered.
Llori et al., 2015 [27]	Predefined and population-dependent	10 population-dependent components (sex-specific tertiles for every component) and three predefined components for alcohol (non-drinkers, moderate, heavy drinkers) and aspirin/NSAID medication (use, non-use).	All dietary components were divided into three tertile values. For antioxidant components: two points were awarded for high-level exposure, one point for intermediate, and 0 for low predominance of antioxidants. The score was reversed for pro-oxidant components. More points were awarded to antioxidant categorical variables (non-drinkers and NSAID use), and fewer points to pro-oxidant categories.	The overall OBS ranged between 0 and 26 points. The score was divided into quartiles.	Questionnaire-based (FFQ) dietary components were considered. Energy adjustment was not considered. Smoking was excluded from the original OBS score because it is a well-known risk factor for CKD.
Wang et al., 2017 [28]	Predefined and population-dependent	Similar OBS components as Dash et al., 2013 [13]: 11 population-dependent dietary components (three categories for every component) and four non-dietary components for smoking, alcohol intake, obesity, and physical activity.	The OBS was built using the weighted method as described by Dash et al., 2013 [13], but with different scoring points. All dietary components were divided into three groups. For antioxidant components: two points were awarded for high-level exposure, one point for intermediate, and 0 for low predominance of antioxidants. The score was reversed for pro-oxidant components. More points were awarded to antioxidant categorical variables (non-smokers, non-drinkers, non-obese and physically active), and fewer points to pro-oxidant categories.	The overall OBS ranged between 0 and 30 points. The score was divided into two or three equal groups (low vs. high and low, intermediate and high).	Questionnaire-based (FFQ) dietary components were considered. Dietary components were adjusted for total energy intake. Nutrient values included dietary and supplemental sources.
Cho et al., 2017 [29]	Predefined and population-dependent	6 population-dependent components (sex-specific quartiles), including four dietary and two non-dietary components (BMI and physical activity), and two non-dietary predefined components for smoking (never, former, current), and alcohol (levels of intake).	For antioxidant components: three points were awarded for high-level exposure, one or two points for intermediate, and 0 for low predominance of antioxidants. The score was reversed for pro-oxidant components. More points were awarded to antioxidant categorical variables (non-smokers, non-drinkers), and fewer points to pro-oxidant categories.	The overall OBS ranged between 0 and 24 points. The score was divided into five categories.	Questionnaire-based (24-h recall) dietary components were considered. Energy adjustment was not considered.
Lee et al., 2017 [30]	Predefined and population-dependent	5 population-dependent components including four dietary components and one lifestyle factor (tertiles) and two predefined components for smoking (never, former, current) and alcohol intake (levels of alcohol intake).	For antioxidant components: three points were awarded for high-level exposure, one or two points for intermediate, and 0 for low predominance of antioxidants. The score was reversed for pro-oxidant components. More points were awarded to antioxidant categories (non-smokers, non-drinkers), and fewer points to pro-oxidants. Three weighting methods were applied: equal weights, weights based on the association with MetS components and weights estimated by Principal Component Analysis.	The overall OBS ranged between 0 and 14 points. The score was divided into quartiles.	Questionnaire-based (24-h recall) dietary components were considered.

BMI: Body Mass Index; CKD: Chronic Kidney Disease; CPS-II: Cancer Prevention Study II; FFQ: Frequency Food Questionnaire; FIP: F2-isoprostanes; FOP: Fluorescent Oxidative Products; MAP study: Markers of Adenomatous Polyps study; MetS: metabolic syndrome; MPC study; Markers of Prostate Cancer study; mtDNA: mitochondrial DNA copy number; NSAID: Non-Steroidal Anti-Inflammatory Drug; OBS: Oxidative Balance Score. Higher OBS values reflect a predominance of antioxidant exposure in almost all OBSs.

**Table 6:** Rationale for the inclusion of some components in *a priori* OBS in relation to OS.

Dietary, Biomarkers, Food, Lifestyle Factors, and Medication Components	
Antioxidants	
**Vitamin C** [31,32]	Antioxidant that scavenges ROS and RNS Prevention of lipid peroxidation Regeneration of α-tocopherol
**Total Carotenoids, Lutein,** **β-carotene, Lycopene,** **β-cryptoxantin, Zeaxantin** [33,34,35,36,37,38]	Deactivators of singlet oxygen and lipid peroxidation Generation of free radical at high oxygen concentration Synergistic antioxidants in biological membranes inhibiting lipid peroxidation Activation transcription factors of antioxidant enzymes Induce the expression of genes encoders for the synthesis of some of the antioxidant enzymes
**Vitamin E** [39]	Lipophilic antioxidant, suppressor of the oxidative damage of polyunsaturated fatty acids present in lipoproteins, biological membranes, and tissues, through the elimination of free radicals such as the radical peroxide Protection of the cell membrane, as well as of various subcellular membranes, against the effects of lipid peroxidation Inhibition of lipid peroxidation in biological membranes Protection against the oxidation of LDL-cholesterol Prevention against risk factors or diseases initiated or promoted by ROS and RNS
**Flavonoids** [40,41,42]	Donation of hydrogen to free radicals Prevention of the formation of free radicals, metal chelators Inhibition of expression, synthesis or activity of pro-oxidant enzymes Induce the expression of genes encoders for the synthesis of some of the antioxidant enzymes
**Glucosinolates** [43,44,45]	Sensitive to induction of electrophiles such as omega-3 PUFAs and hemoxygenase-1, which catalyzes heme to biliverdin and the induction of glutathione peroxidase Induce the expression of genes encoding the synthesis of some of the antioxidant enzymes
**Minerals: Se and Zn** [46,47,48]	Cofactors of enzymes involved in the endogenous antioxidant system that interrupt cellular oxidative processes
Prooxidants	
**Total Fats**	Intake of lipids can contribute to oxidative stress through lipid peroxidation
**PUFAS** [54,55,56,57,58,77]	Increase the formation of lipid peroxides that contribute to oxidative stress PUFAs are involved in the regulation of inflammatory activity. Fatty acids n-6 are pro-inflammatory and fatty acids of n-3 are anti-inflammatory
**SFA** [53]	Oxidative DNA damage
**Iron/Ferritin** [59,60,61,62]	Association with oxygen transport; can catalyze oxidative reactions in the formation of free radicals Oxidative damage to lipid membranes (atherogenesis promotion) by increasing the formation of free radicals and oxidative stress that induces the peroxidation of proteins and lipids Possible intensification of oxidative stress by catalyzing the production of highly reactive hydroxyl radicals through the Haber-Weiss reaction
**Red Meat** [62]	Possible intensification of oxidative stress mediated by iron intake contained in red meat Promotion of atherogenesis
**Lifestyle factors**	
Antioxidants	
**Physical Activity** [63,64]	Increase in the adaptive response to oxidative stress by activating the cellular antioxidant signaling systems and improving the expression of antioxidant enzymes
Prooxidants	
**Alcohol** [65,78,79]	Possible increase in ROS generation and increase of inflammatory processes Induction of OS by oxidation of ethanol to acetaldehyde, which can lead to the production of ROS and RNS, oxidation of nucleic acids and decrease in the activity of antioxidant enzymes
**Smoking Status** [66,67,68,69,70]	Exogenous prooxidant: increased oxidative stress and oxidative imbalance in cellular tissues The increase of the OS load of inhaled tobacco smoke could increase through the secondary release of oxygen radicals from the inflammatory cells Increase in markers of oxidative stress in blood and tissues
**BMI, Obesity** [71]	Related to increased ROS markers Leads to redox imbalance and increased lipid peroxidation, which can lead to ROS production
**Medication**	
Antioxidants	
**Aspirin** [72,73,74,75]	Inhibition of ROS production in human endothelial cells exposed to oxidized LDL-cholesterol Regulation of ROS and RNS to reduce inflammation and cell damage
**NSAIDs** [72,73,74,75]	Regulation of ROS and RNS to reduce inflammation and cell damage

BMI: Body Mass Index; DNA: Deoxyribonucleic acid; NSAID: Non-Steroidal Anti-Inflammatories; LDL-cholesterol: Low-density lipoproteins-Cholesterol; OS: Oxidative Stress; PUFA: Polyunsaturated fatty acids; ROS: Reactive Oxygen Species; RNS: Reactive Nitrogen Species; SFA: Saturated Fatty Acids.

**Table 7:** Main characteristics of studies analyzing the association between *a priori* Oxidative Balance Scores and health outcomes.

Author (s), Year	Country, Population (*N*), Year of Recruitment, Age at Entry	Study, Design, Follow-up Time (Years)	Main Outcome	Covariables in Adjusted Model	OR/RR/HR (95 % CI) ^a^, Multivariable Adjusted
Van Hoydonck et al., 2002 [9]	Belgium 2814 male smokers 1980–1984; 25–74 years	BIRNH study Cohort study (10 years)	All-cause mortality Cancer mortality CVD mortality	Age, educational level, BMI, total energy intake and smoking (pack-years)	RR for high vs. low OBS: 1.44 (1.13–1.82) for all-cause mortality 1.62 (1.07–2.45) for cancer mortality 1.31 (0.86–2.00) for CVD mortality (not significant)
Goodman et al., 2007 [10]	USA MAP study 170 cases and 230 controls 1995–1997; ≥50 years MPC study 89 cases and 197 controls 1994–1996; ≥50 years	MAP and MPC studies Case-control studies	Adenomatous polyps Prostate cancer	Age, sex, race, energy intake	MAP study (adenomas) OR for high vs. low OSS = 0.45, (0.21–0.99) MPC study (prostate cancer) OR high vs. low OSS = 0.28, (0.28–0.82)
Goodman et al., 2008 [15]	USA 574 cases, 1227 endoscopy controls and 550 community controls 1991–1994; 30–74 years	Minnesota Digestive Healthcare Case-control study	Colorectal adenomas	Age, sex, hormone therapy, race, education, family history of colorectal cancer, energy intake, BMI, alcohol consumption, calcium, vitamin D, folic acid, red meat, multivitamin and dietary fiber	OR for high vs. low OBS 0.19 (0.06–0.57; *p*-trend < 0.001) for endoscopy controls 0.24 (0.06–0.94; *p*-trend = 0.002) for community controls
Goodman et al., 2010 [18]	USA MAP study 111 cases and 115 controls 1995–1997; ≥50 years MPC study 97 cases and 226 controls 1994–1996; ≥50 year	MAP and MCP studies Case-control studies	Colorectal adenomas Prostate cancer	Age, race, total energy intake, blood cholesterol, BMI, and family history of prostate cancer or colorectal cancer In addition, the MAP study controlled for sex, and for hormone replacement therapy among women	OBS (continuous, per unit increment) OR = 0.90 (0.83, 0.97) in both studies MAP study (adenomas) OR high vs. low OBS = 0.34 (0.13–0.88) MPC study (prostate cancer) OR high vs. low OBS = 0.34 (0.14–0.86)
Agalliu et al., 2011 [16]	Canada 661 cases and 1864 subcohort 1992–2003; 66.2 years (mean cases) and 69.3 years (mean subcohort)	CSDLH study Case-cohort (4.3 vs. 7.7)	Prostate cancer	Age, race, BMI, physicalactivity, and education	No association HRs high vs. low OBS across quintiles: 1.02, 1.03, 0.97 and 1.01; *p*-trend = 0.71. No association by aggressiveness types
Slattery et al., 2012 [19]	Utah, USA KPMCP Colon cancer: 1555 cases and 1956 controls: 1991–1994 Rectal cancer: 974 cases and controls; 1997–2001; 30–79 years	Case-control study	Colon cancer Rectal cancer	Total energy intake in analyses with dietary variables	OR high vs. low OBS: 0.52 (95% CI: 0.41–0.66) for colon cancer 0.49 (95% CI: 0.35–0.70) for rectal cancer
Geybels et al., 2012 [17]	The Netherlands 3451 cases and 2191 subcohort 62.8 years (mean cases)	NLCS study Case-cohort study 17.3 years	Prostate cancer	Age, smoking intensity and duration	HR for high vs. low OBS: 1.16 (95% CI: 0.98–1.37) No association by stage of the disease
Dash et al., 2013 [13]	USA 789 cases and 1500 controls 1991–2002; 30–74 years	CPRU study, MAP I study, MAP study Case-control studies	Colorectal adenomas Validation study with FIP levels	Age, sex, education, family history of colorectal cancer, aspirin, nonsteroidal anti-inflammatory, calcium, vitamin D, folate, fiber, energy intake, cumulative estrogen exposure, excluding oral contraceptive use and use of menopausal hormone therapy	OR for high vs. low OBS ranged from 0.38–0.54 for the 4 OBS (all were statically significant). OR-equal weight = 0.54 (0.43–0.69) OR-lit review = 0.45 (0.35–0.58) OR-*a posteriori* = 0.38 (0.29–0.49) OR-Bayesian = 0.45 (0.35–0.58) Negative association between the OBS and FIP
Labadie et al., 2013 [20]	USA 472 cases and 578 controls 1991–2002 59 years in cases and 54 years in controls	CPRU study; MAP I study, MAP II study Case-control studies	Colorectal adenomas by genetic variants of antioxidant genes (SOD2, CAT, GSTP1)	Age, sex, hormone therapy, family history of colorectal cancer, body composition, energy intake, physical activity, calcium, fiber, red meat, vitamin D (dietary + supplemental)	The OBS was not associated with colorectal adenoma risk by the genetic polymorphisms, individually or in combined gene scores
Kong et al., 2014 [11]	USA 139 cases and 201 controls 1991–2002 56.9 years in cases and 55.9 years in controls	MAP I MAP II Case-control studies	Colorectal adenoma Validation study with FIP, FOP and CRP levels	Age, race, sex, BMI ^d^, energy intake, plasma cholesterol, family history of colorectal cancer, hormone replacement therapy, fiber, physical activity, study (MAP I or MAP II)	OR for high vs. low OBS = 0.39 (0.17–0.89) Biomarkers associations between the OBS and CRP (negative), FOP (positive) and FIP (negative)
Slattery et al., 2014 [21]	USA Hispanic: 2111 cases and 2597 controls Non-Hispanic: 1481 cases and 1586 controls 1995–2007; 25–79 years	Breast Cancer Health Disparities study Case-control	Breast cancer	Age, study center, BMI in referent year, parity, genetic admixture	OR for high vs. low OBS = 0.74 (0.64–0.84)
Lakkur et al., 2014a [22]	USA 43,325 men and 3386 cases 1999–2007; 70 years (mean)	CP Study II Nutrition Cohort Cohort study (8 years)	Prostate cancer Aggressive disease Non-aggressive disease	Age, energy intake, calcium, vitamin D and folate intake, race, education, family history of prostate cancer, cholesterol lowering drug use, finasteride use, history of prostate cancer screening	HR for high vs. low OBS: HR-equal weight = 1.17 (1.04–1.32) HR literature-weight = 1.15 (1.03–1.30) *p* for interaction by aggressiveness types > 0.05
Lakkur et al., 2014b [12]	USA 321 participants; 25–74 years	SRSH study Cross-sectional	FIP, FOP, mtDNA	Age, sex, BMI, and race/origin	Negative association with FIP (OR high vs. low OBS = 0.04; 95% CI: 0.01–0.17) but positive with FOP (OR high vs. low OBS = 5.64; 95% CI: 2.35–13.54). The association for mtDNA copy number was unstable. Varying associations with FIP, FOP or mtDNA.
Dash et al., 2015 [23]	USA 80,063 1999–2009; 68–70 years	CP Study II Nutrition Prospective cohort study (10 years)	Colorectal cancer	Age, sex, education, family history of colorectal cancer in a first-degree relative, colorectal cancer screening, nonsteroidal anti-inflammatory, calcium, vitamin D, energy intake, and hormone replacement therapy	RR for high vs. low quartile: OBS-equal weight 0.59 (0.49–0.70) OBS-literature review 0.60 (0.50–0.73) OBS-*a posteriori* 0.47 (0.39–0.57) OBS Bayesian 0.50 (0.41–0.61)
Kong et al., 2015 [24]	USA 21,031 2003–2007, ≥45 years	REGARDs study Prospective cohort study (5.8 years)	All-cause mortality Cancer mortality Non-cancer mortality CVD mortality	Age, sex, race, SES, region, BMI, energy intake, and physical activity	HR for high vs. low OBS: 0.70 (0.61–0.81) for all-cause mortality; 0.50 (0.37–0.67) for cancer-mortality; 0.77 (0.66–0.89) for non-cancer mortality; Not significant for CVD mortality
Annor et al., 2015 [25]	USA 317 participants; 25–74 years	SRSH study Cross-sectional	Hypertension Oxidative stress markers FIP, FOP and mtDNA	Age, sex, education, and race/origin	OR for high vs. low OBS = 0.17 (0.79–0.96) OR per 1 unit increase in OBS = 0.87 (0.79–0.96) Negative correlation between OBS and FIP, but positive for FOP
Lakkur et al., 2015 [26]	USA 19,825 participants 2003–2007; ≥45 years	REGARDs study Cohort study (4 years)	CRP Waist circumference LDL-cholesterol HDL-cholesterol Total cholesterol Serum albumin Triglycerides	Age, sex, energy intake, BMI, race, educational level, region, and physical activity	OR for high vs. low OBS: 0.50 (0.38–0.66) for CRP; 0.50 (0.36–0.71) for waist circumference; 0.75 (0.58–0.98) for LDL-cholesterol. 0.48 (0.28–0.83) for HDL-cholesterol in women and 1.63 (1.09–2.45) in men No significant associations for serum albumin, total cholesterol and triglycerides.
Ilori et al., 2015 [27]	USA 19,461 participants (90 incident ESRD cases; 2519 prevalent albuminuria; 1957 prevalent CKD) 2003–2007; ≥45 years	REGARDs study Cohort study (3.5 years) and cross-sectional study for baseline markers of chronic kidney disease	ESRD CKD Albuminuria Macroalbuminuria Incident ESR	Age, sex, race, region and calories, BMI, smoking, waist circumference, physical activity, education, income, SBP, DBP, total cholesterol, CAD, diabetes and statin medications	OR/HR for high vs. low OBS: 0.67 (0.49–0.92) for macroalbuminuria; 0.79 (0.67–0.92) for CKD OR per 5-units OBS: 0.83 (0.72–0.96) for macroalbuminuria; 0.90 (0.84–0.97) for CKD Not significant for ESRD and albuminuria
Wang et al., 2017 [28]	USA 488 cases and 604 controls 1991–2002; 30–74 years	CPRU, MAP I, MAP II Case-control study	Interaction between based excision repair genes (BER) in genetic scores and OBS with colorectal adenoma risk	Age, sex, family history of colorectal cancer in a first degree relative, NSAID use, energy intake, fiber, circulating 25-OH-vitamin D3 concentration	OR for high weighted BER score and low OBS = 2.19 (1.19–3.99); OR for low weighted BER score and low OBS = 1.07 (0.61–1.93); OR for high weighted BER score and high OBS = 1.38 (0.75–2.53) *p*-value for interaction = 0.42
Cho et al., 2017 [29]	Korea 2087 men and 2071 women 2010–2011; 19–65 years	KNHANES-V study Cross-sectional study	GGT	Age, energy intake, fasting plasma glucose, total cholesterol, SBP, and alanine aminotransferase	OR for high vs. low OBS = 0.05 (0.01–0.19) for men and 0.27 (0.09–0.78) for women (*p* for trend < 0.01).
Lee et al., 2017 [30]	Korea 6414 subjects 2001–2002; ≥40 years	KARE cohort study Cross-sectional study	MetS Inflammatory markers: CRP	Age, geographic area, sex, and BMI	OR for high vs. low OBS: for equal weights = 0.65 (0.51–0.83); for beta-coefficients weights = 0.56 (0.76–0.41); for principal components weights = 0.55 (0.40–0.75) No association between the OBS with any of the MetS components except for waist circumference (β = −0.98; *p*-value =< 0.01).

Antioxidant genes: SOD2 (superoxide dismutase), CAT, GSTP1; BER GRS: Base Excision Repair Genetic Risk Scores; Biomarkers: CRP, FIP, FOP: C-Reactive Protein, F2-isoprostanes, Fluorescent Oxidative Products; BIRNH study: Belgian Interuniversity Research on Nutrition and Health study; BMI: Body Mass Index; CAD: Coronary Artery Disease; Cholesterol: HDL (high density lipoproteins), LDL (low density lipoproteins); CKD: Chronic Kidney Disease; CPRU study: Cancer Prevention Research Unit. CP study II Nutrition Cohort: Cancer Prevention Study II Nutrition Cohort; CSDLH study: Canadian Study of Diet, Lifestyle and Health cohort; CVD: cardiovascular disease; DBP: Diastolic Blood Pressure; ESRD: End Stage Renal Disease; EPX rs2302313: eosinophil peroxidase; GGT: γ-glutamyltransferase; KARE study: Korea Association Resource study; KNHANES-V study: Korea National Health and Nutrition Examination survey; KPMCP: Kaiser Permanente Care Program of Northern California; GRS: Genetic Risk Scores; MAP study: Markers of Adenomatous Polyps study; MetS: Metabolic syndrome; MPC study: Markers of Prostate Cancer study; MPO rs2243828: myeloperoxidase; NA ancestry: Native American ancestry; NLCS study: Netherlands cohort study; NOS2A: nitric oxide synthase; NSAID: nonsteroidal anti-inflammatory drug; OBS: Oxidative Balance Score; OSS: Oxidative Stress Score; SBP: systolic blood pressure; SEER summary stage: Surveillance Epidemiology and End Results; SES: Socioeconomic status; SRSH study: Study of Race, Stress, and Hypertension; REGARDs study: Reasons for Geographic and Racial Difference in stroke study; USA: United States; WBC count: waist B circumference count. ^a^ OR: Odds Ratio, RR: relative risk and HR: hazard ratio was calculated compared the lowest OBS category vs. the highest OBS category (categories defined as tertiles, quartiles or quintiles).

## Abbreviations

BER GRS	Base Excision Repair Genetic Scores
BIRNH study	Belgian Interuniversity Research on Nutrition and Health study
BMI	Body Mass Index
CAD	Coronary Artery Disease
CI	Confidence Interval
CKD	Chronic Kidney Disease
CPRU study	Cancer Prevention Research Unit study
CP study II Nutrition Cohort	Cancer Prevention Study II Nutrition Cohort
CRP	C-Reactive Protein
CSDLH cohort	Canadian Study of Diet, Lifestyle and Health cohort
CVD	Cardiovascular disease
DBP	Diastolic Blood Pressure
DNA	Deoxyribonucleic acid
ESRD	End-Stage Renal Disease
FFQ	Food Frequency Questionnaire
FIP	F2-isoprostanes
FOP	Fluorescent Oxidative Products
GCS	Glucosinolates
GGT	γ-glutamyltransferase
GRS	Genetic Risk Score
HDL-cholesterol	High-Density Lipoproteins-Cholesterol
HR	Hazard Ratio
KARE study	Korea Association Resource study
KNHANES-V	Korea National Health and Nutrition Examination survey study
KPMCP	Kaiser Permanente Care Program of Northern California
LDL-cholesterol	Low-Density Lipoproteins-Cholesterol
MAP study	Markers of Adenomatous Polyps study
MetS	Metabolic syndrome
MPC study	Markers of Prostate Cancer study
mtDNA	Mitochondrial DNA copy number
NOS2A	Nitric oxide synthase
NSAID	Non-steroidal anti-inflammatory drug
OBS	Oxidative Balance Score
OR	Odds Ratio
OS	Oxidative stress
OS biomarkers	Oxidative stress biomarkers
OSS	Oxidative Stress Score
PUFAS	Polyunsaturated fatty acids
SBP	Systolic blood pressure
SEER summary stage	Surveillance Epidemiology and End Results summary stage
SES	Socioeconomic status
SFA	Saturated Fatty Acids
SRSH	Study of Race, Stress, and Hypertension
REGARDs study	Reasons for Geographic and Racial Difference in stroke study
RNS	Reactive Nitrogen Species
ROS	Reactive Oxygen Species
RR	Relative Risk
US	United States
WBC count	Waist B circumference count
